# Evolution of Manufacturing Defects of 3D-Printed Thermoplastic Composites with Processing Parameters: A Micro-CT Analysis

**DOI:** 10.3390/ma16196521

**Published:** 2023-09-30

**Authors:** Hantai Wu, Xinyu Chen, Shuaiheng Xu, Tian Zhao

**Affiliations:** 1 Beijing Key Laboratory of Lightweight Multi-Functional Composite Materials and Structures, Institute of Advanced Structure Technology, Beijing Institute of Technology, Beijing 100081, China; 3220212078@bit.edu.cn (H.W.);; 2 Beijing Institute of Astronautical System Engineering, Beijing 100076, China

**Keywords:** 3D printing, fiber-reinforced thermoplastic composites, process parameters, micro-CT, microstructural analysis

## Abstract

Owing to the melting and healing properties of thermoplastic resin, additive manufacturing or 3D printing is considered one of the most promising technologies for fiber-reinforced thermoplastic composites. However, manufacturing defects are still the main concern, which significantly limits the application of 3D-printed composite structures. To gain an insight into the effects of different processing parameters on the typical manufacturing defects, a micro-scale analysis was carried out via Micro-CT technology on the 3D-printed continuous carbon fiber-reinforced polylactic acid (PLA) composite specimens. The bias distribution of the fiber in the deposited filament was found. Moreover, when the feed rate of the filament was reduced from 100% to 50%, the a/b value was closer to 3.33, but the porosity increased from 7.077% to 25.352%. When the layer thickness was 0.2 mm, the increased nozzle pressure reduced the porosity but also increased the risk of fiber bundle breakage. The research provides an effective approach for analyzing the micro-structure of 3D printed composite structures and thus offers guidance for the processing control.

## 1. Introduction

Benefiting from their excellent mechanical properties and cost-effective manufacturing methods, thermoplastic composites (TPCs) have gained an increasing attraction in many industrial fields, such as aerospace, automotive and medical engineering, etc. [1,2,3]. Thermoplastic resin can be heated and melted when given an increased temperature and retains the original properties after consolidation [4]. Therefore, a number of rapid manufacturing techniques, e.g., automatic fiber laying (AFP) [5], automatic tape layering (ATL) [6], compression molding [7], pultrude molding [8], etc., can be applied on TPCs to form structures with complex configurations within a short processing time [9]. Among them, additive manufacturing (AM), or 3D printing, is considered one of the most advanced techniques, which significantly improves structural design flexibility [10,11]. Three-dimensional printing technology is based on 2D slices deposited layer upon layer into a 3D object, without molds, and reducing material waste [12]. Extensive research has been conducted on the 3D printing technique of thermoplastic resin and their composites [13,14], which greatly promotes the development of this manufacturing technology.

Nevertheless, the development of 3D printing techniques for continuous fiber-reinforced thermoplastic composites (CFRTPCs) has been slow. For example, Fused Deposition Modeling (FDM) is the most widely used and promising 3D printing technique for CFRTPCs [15]. In the FDM technology, the continuous fiber bundle is fed into the extrusion head, impregnated by a heated thermoplastic resin, and then deposited onto the printing platform via a small diameter nozzle to print the part layer by layer [16], whereas typical defects always exist within the FDM technology of CFRTPC structures, which prevents further application of this technology. Rahmatabadi et al. [17] studied the compression, bending, and tensile properties of PVC manufactured by FDM and demonstrated that the presence of high-density cavities in FDM-manufactured parts is the source area of crack growth and failure. Tian et al. [18] systematically studied the influence of 3D printing process parameters on the interface and properties of printed composite materials and found that temperature and pressure are the key parameters of the molding process. By optimizing the process parameters, the maximum bending strength of 3D printing continuous fiber-reinforced PLA with 27% fiber content reached 335 MPa, and the bending modulus was 30 GPa. Geng et al. [19] found that melt pressure (controlled by printing speed and extrusion speed) directly affects the surface morphology and extrusion diameter of the filament in the FDM process of PEEK. Therefore, it can be inferred that the mechanical properties of FDM technology for CFRTPCs are significantly influenced by the microscopic defects introduced during the printing process. Simultaneously, ensuring the reliability of printed filaments is crucial to meet the demands of manufacturing complex 3D configurations.

To overcome these problems, many scholars have carried out relevant investigations. Zhang et al. [20] studied the effect of layer thickness on the void distribution of continuous carbon fiber-reinforced PLA composites. They found that decreasing the layer thickness effectively improved the ironing force, thus minimizing the formation of void age and improving the surface quality of the printed specimens. Shuto et al. [21] studied the influence of nozzle temperature on the porosity and filament deformation and concluded that nozzle temperature had little influence on porosity, while filament area fraction was higher at both ends of the printable temperature range. Liao et al. [22] used high-resolution X-ray microscopy to quantitatively count the number of voids in different printing directions and obtained results that the void volume of the sample with the 0° printing direction was much higher than that with the printing path of 45/−45 or 0/90.

High-resolution X-ray micro-computed tomography (denoted as Micro-CT hereafter) is a very interesting non-destructive testing technology that can be used to reconstruct internal structural details, hence becoming an effective method that is increasingly used for the quantitative analysis of manufacturing defects within composite structures [23,24]. Yamamoto et al. [25] observed the inside of the samples printed under different path-planning algorithms by Micro-CT and suppressed the voids in the actual printing process by adjusting the parameters of the algorithm. He et al. [26] quantitatively measured the number of voids in 3D-printed continuous fiber-reinforced Polyamide6 specimens via Micro-CT. Zhang et al. [27] observed the Micro-CT images of the printing-induced defects such as fiber wrinkling, twisting, and folding at different turning angles (30°~180°). Yu et al. [28] used Micro-CT to characterize the position, length, and direction of short-fiber in basalt–fiber-reinforced PLA composites and statistically analyzed the changes in the number of two types of voids under the influence of different fiber contents.

In this study, the microstructure characteristics of CFRTPCs prepared by FDM technology were characterized by Micro-CT, focusing on the morphology and porosity of the printed filaments. The deformation of filaments, void volume distribution, and void distribution in space under different parameters were quantified, and the influence of printing parameters on the morphology and printing defects of the specimen was discussed. Provided a reference for the parameter selection and process optimization during the preparation of CFRTPCs by FDM technology.

## 2. Materials and Experiments

### 2.1. Materials and Printing Equipment

**Figure 1:**
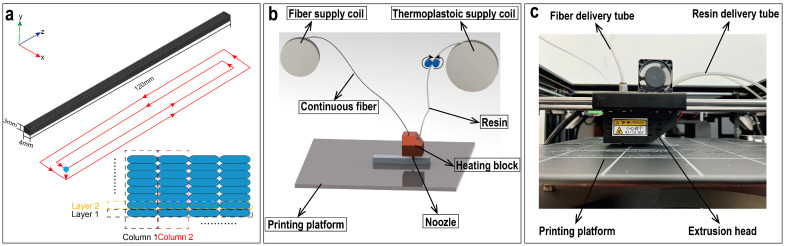
(**a**) Schematic of the size and printing path of the printed composite specimen; (**b**) simulation diagram of the in situ fusion technology; (**c**) diagram of the printing equipment used in the experiment.

In this study, a T300-1 K carbon fiber bundle, provided by Toray Co., Tokyo, Japan, with an average single-fiber diameter of 7 μm was used as the reinforcement. PLA filament with a diameter of 1.75 mm, produced from PolyLite™., Suzhou, China, was used as the thermoplastic resin. All specimens were fabricated using a COMBOT-200 FDM 3D printer provided by Shaanxi Fibertech Technology Development Co., Ltd., Shaanxi, China. The nozzle diameter was 1.0 mm. The geometry of the 3D-printed sample was modeled and then was cut into thin layers using the slicing software. The main size and printing path of the specimen are schematically shown in Figure 1a. The filaments along the X and Y directions were defined as the ‘layer’ and the ‘column’, respectively. After slicing, the G-code [29] was saved and transferred to the FDM printer to carry out the printing process. 

The 3D printer used in situ fusion technology, which means during the printing process, the fiber bundle and resin filament were separately transported through different fed tubes and were mixed in the heating block. The moving extrusion head deposited the filament on the unheated printing platform in accordance with the set print path, as illustrated in Figure 1b. After a certain layer was deposited, the print head was raised to continue to deposit the next layer, and the printing path of all layers was the same.

### 2.2. Process Conditions and Parameters

In this research, a series of typical printing parameters, i.e., nozzle temperature (T), printing speed (S), Feed rate of the filament (F) (related to the unit volume of resin fed into the printing head), and layer thickness (H) were selected to analyze their effects on the quality of the as-manufactured specimens. The design matrix of the experimental study is listed in Table 1.

### 2.3. Characterization Methods

**Figure 2:**
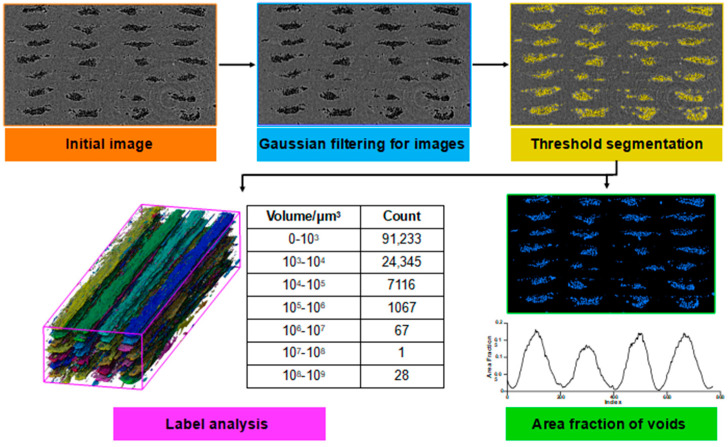
Steps of void extraction and quantitative analysis.

The filament morphologies and void contents of the 3D-printed composite specimens were acquired to assess the effects of different parameters on the quality of the specimens. Cross-sectional slices of the specimens were gathered via a Diondo-D2 Micro-CT scanner (Diondo, Hattingen, Nordrhein-Westfalen, Germany) with a 90 kV accelerating voltage and a 90 μA current. The Micro-CT resolution was approximately 5 μm. Each specimen was rotated in equal increments over 360°, and 1860 projections were collected. Subsequently, 2D slices were imported into Avizo (FEI, Hillsboro, OR, USA) software, and 3D models of the specimens were reconstructed. The analysis process is shown in Figure 2.

## 3. Results and Discussion

### 3.1. Morphology Characterization of Filaments

**Figure 3:**
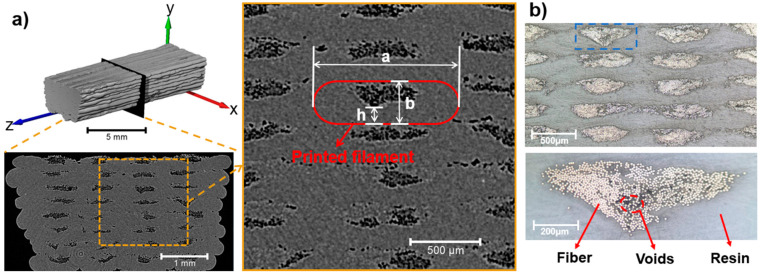
(**a**) Reconstructed cross-sectional image of the printed specimen T230 based on Micro-CT scan, magnification of the part inside the rectangular frame: the profile of the deposited filament is outlined by the red line; and (**b**) optical microscopy images of the specimen T230 at different magnifications.

Cross-sectional images of the T230 printed specimen reconstructed from the CT scanning results and the light microscopy are illustrated in Figure 3. Due to the similar density of PLA resin and carbon fiber, the colors of these two are similar in the CT scan results. Therefore, the gray pixels correspond to the fiber and resin, and the black pixels correspond to the voids. Two interesting points were observed with respect to filament morphology. Firstly, the cross-sectional area of the deposited filament is shown as a rounded rectangle instead of a circular shape. This is likely a result of the contact pressure of the nozzle during the printing process. More importantly, an offset distribution at the top part of the entire filament was found for the voids; at the same time, the fiber bundle is concentrated in the top area of the filament. The same phenomenon was observed in [30].

**Figure 4:**
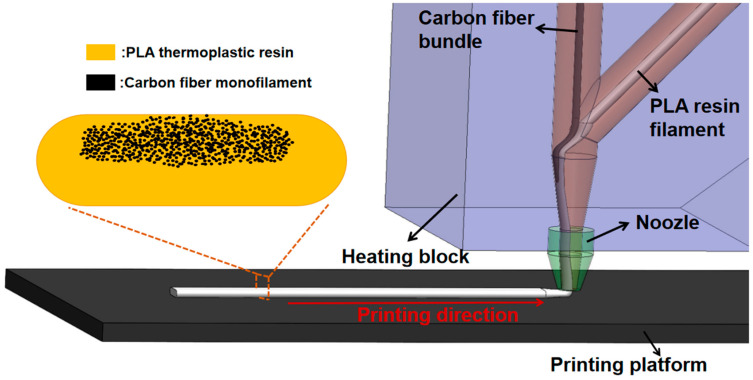
A schematic diagram of the spatial distribution of the fiber bundle and PLA resin filament in the heating block tube and nozzle during the printing process.

A possible explanation for the offset distribution of the fiber bundles in the entire filament is illustrated in Figure 4. During the printing process, the unimpregnated dry carbon fiber bundle and PLA thermoplastic resin filament were independently fed from different inlet tubes and mixed in the heating block. The resin tube was distributed in front of the one for the fiber bundle with respect to the printing direction, which formed an initial spatial distribution of these two components. Subsequently, the PLA resin was heated and melted, thus immersing the fiber bundle. Due to the inertia effect of the printing head movement, the fiber bundle constantly laid on the back side of the nozzle, which finally resulted in a concentrated distribution of the fiber bundle on the upper part of the deposited filament.

In order to quantitatively characterize the effects of printing parameters on the filament morphology, three geometrical parameters for the filament were defined: a and b are the length and the width of the rounded rectangle, respectively, and h is the vertical distance from the position where the voids begin to appear to the bottom of the filament, as indicated in Figure 3a. Therefore, the ratios a/b and h/b were selected to characterize the morphology of the deposited filament and the offset magnitude of the fiber bundle during the printing processes with different parameters.

#### 3.1.1. Effect of Printing Parameters on a/b

**Figure 5:**
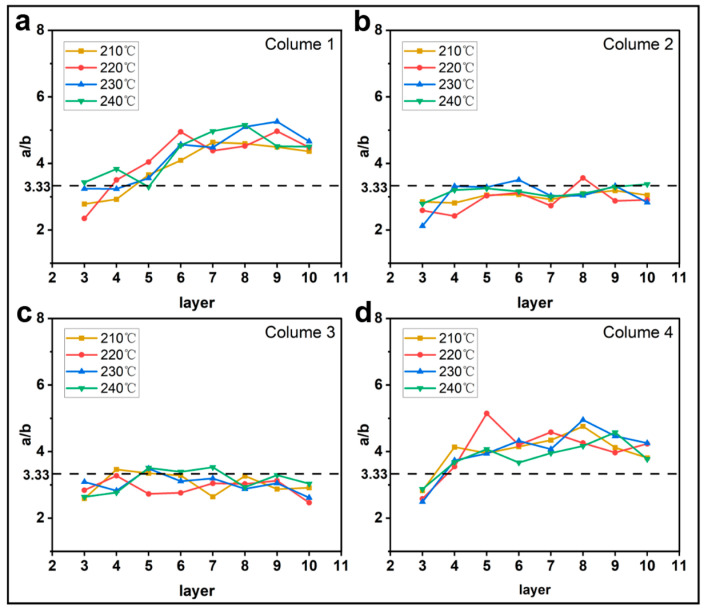
Influence of printing temperature on the filament morphology index a/b. The four sub-diagrams in (**a**–**d**) correspond to the four columns of the printed specimens, respectively (see Figure 1a).

**Figure 6:**
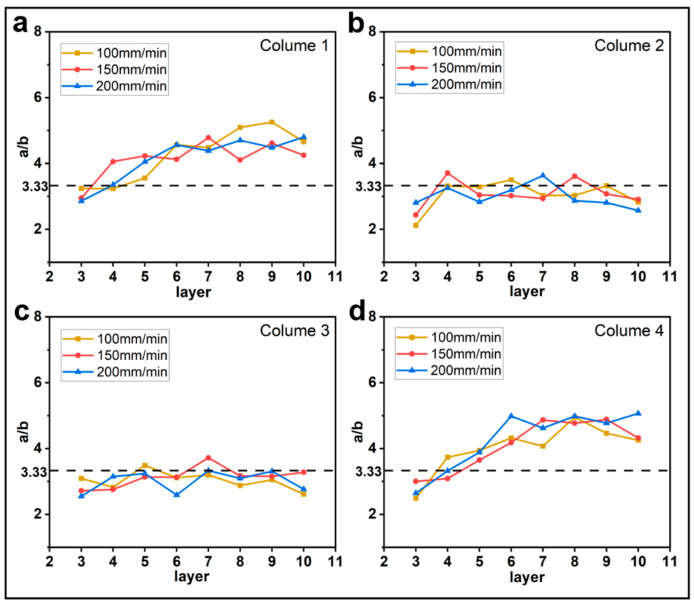
Influence of printing speed on the filament morphology index a/b. The four sub-diagrams in (**a**–**d**) correspond to the four columns of the printed specimens, respectively.

**Figure 7:**
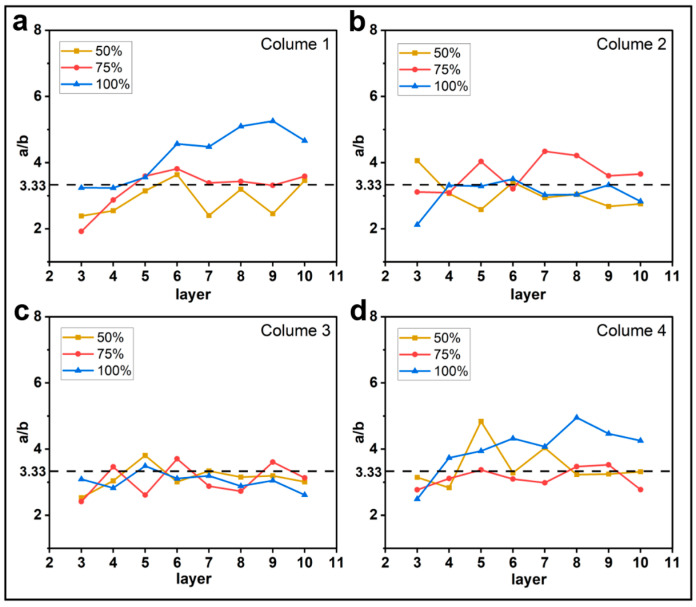
Influence of feed rate of the filament on the filament morphology index a/b. The four sub-diagrams in (**a**–**d**) correspond to the four columns of the printed specimens, respectively.

**Figure 8:**
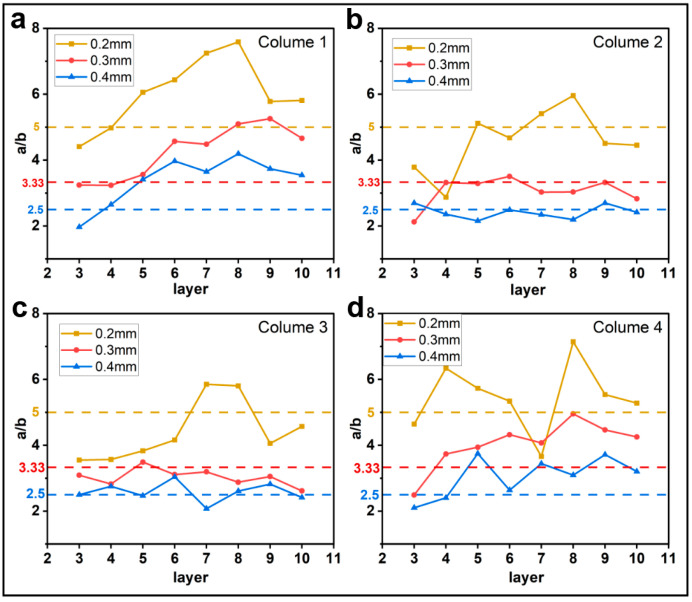
Influence of layer thickness on the filament morphology index a/b. The four sub-diagrams in (**a**–**d**) correspond to the four columns of the printed specimens, respectively.

Figure 5, Figure 6, Figure 7 and Figure 8 show the evolution of the parameter a/b as the layers are superimposed within different columns. The horizontal dotted lines in the figures represent the theoretical values of a/b, which are 5, 3.33, and 2.5 for the layer thicknesses of 0.2 mm, 0.3 mm, and 0.4 mm, respectively. In Figure 5, Figure 6 and Figure 7, it is interesting to find that the ratio a/b of the first and the fourth columns gradually increases, and the major part is greater than the theoretical value, while the curves of the second and third columns basically float around the horizontal dotted line. As shown in Figure 7, when the feed rate of the filament is 50% and 75%, the a/b of each column fluctuates around the theoretical value but with higher amplitudes. This tends to indicate that when the feed rate of the filament is reduced, the shape of the filament is more inclined to the theoretical shape, but the possibility of the filament deformation is also increased. Regarding the layer thicknesses of 0.3 mm and 0.4 mm, the curves show a similar trend. In comparison, the ratio a/b for the thickness of 0.2 mm is obviously higher, and more significant oscillations are observed in the curves, as shown in Figure 8. This is likely due to the fiber breakage, which is caused by the increase in nozzle pressure when the layer thickness is reduced [20].

#### 3.1.2. Effect of Printing Parameters on h/b

**Figure 9:**
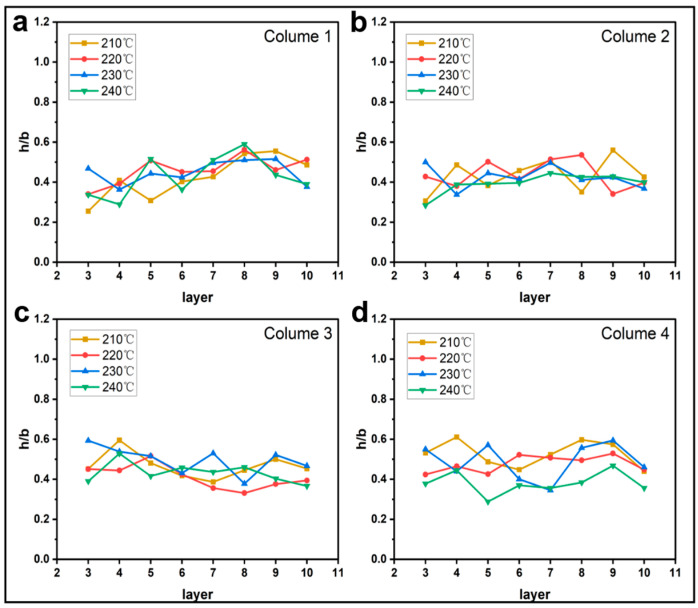
Influence of printing temperature on the filament morphology index h/b. The four sub-diagrams in (**a**–**d**) correspond to the four columns of the printed specimens, respectively.

**Figure 10:**
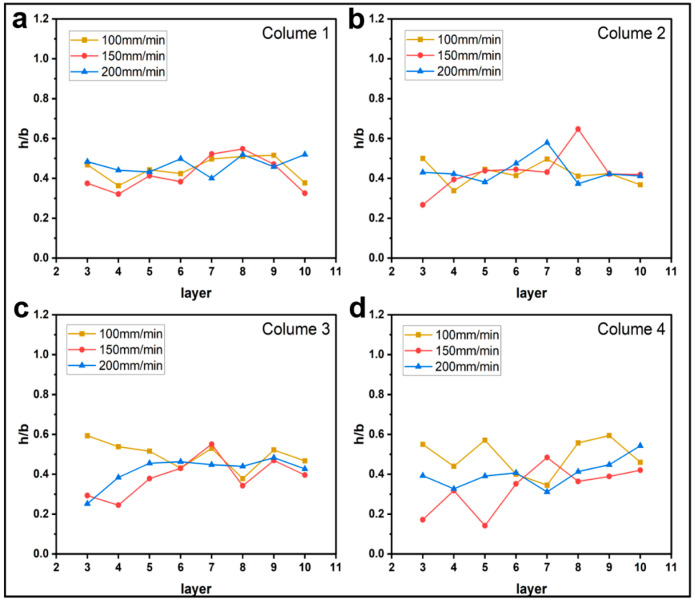
Influence of printing speed on the filament morphology index h/b. The four sub-diagrams in (**a**–**d**) correspond to the four columns of the printed specimens, respectively.

**Figure 11:**
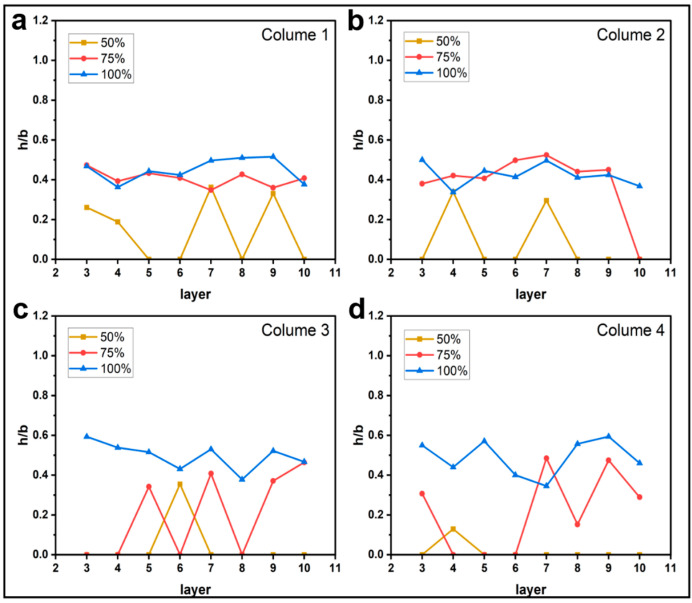
Influence of feed rate of the filament on the filament morphology index h/b. The four sub-diagrams in (**a**–**d**) correspond to the four columns of the printed specimens, respectively.

**Figure 12:**
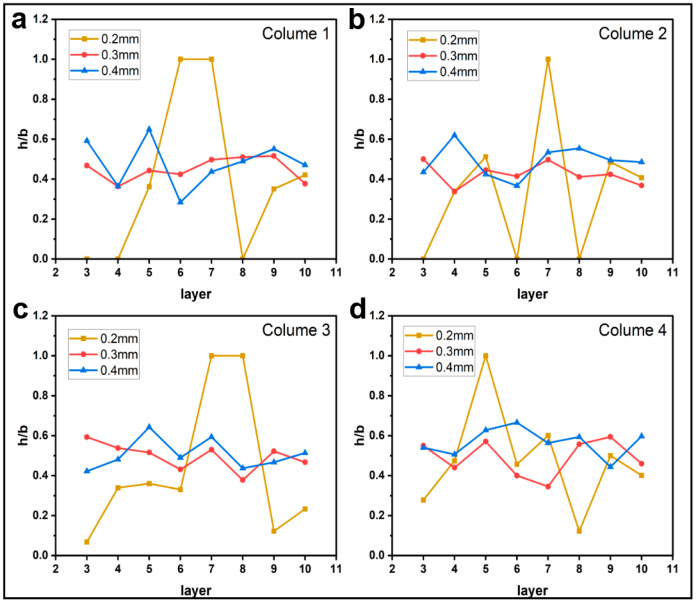
Influence of layer thickness on the filament morphology index h/b. The four sub-diagrams in (**a**–**d**) correspond to the four columns of the printed specimens, respectively.

Figure 9, Figure 10, Figure 11 and Figure 12 plot the variation of parameter h/b as the layers are superimposed. According to Figure 9 and Figure 10, neither printing temperature nor printing speed have an apparent influence on the curve trend. Generally, the curves are constantly leveling at around the ratio h/b of 0.4. In the direction of the thickness of a single deposited filament bundle, the fibers are basically clustered in the upper half area. When the feed rate of the filament decreases, it is found that the value of h/b possibly drops to 0, as shown in Figure 11. This can be explained by the fact that owing to the reduced contents of thermoplastic resin in the nozzle, the fiber bundle has more flexibility and thus is more inclined to disperse throughout the entire filament instead of gathering in the upper part. This possibility is greatly increased when the feed rate of the filament is reduced. Regarding the effects of layer thickness, another extreme case is that the value of h/b is equal to 1 occurs once given a 0.2 mm thickness. This indicates that there are almost no voids in the filament, which is most likely because the fiber bundle was broken in this part, hence the filament was filled with pure resin.

### 3.2. Characterization of Porosity Content

**Figure 13:**
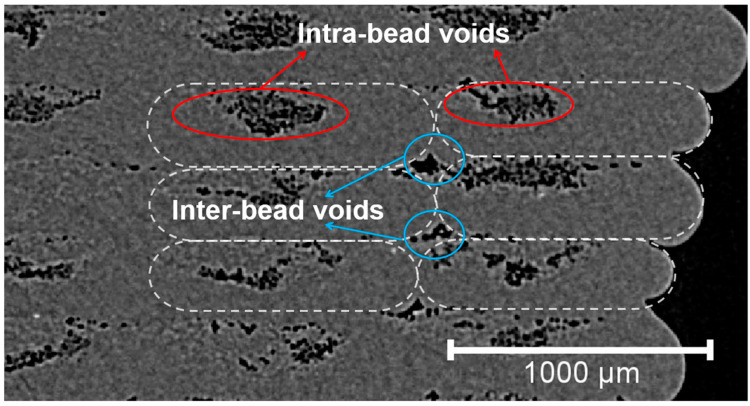
A typical Micro-CT image of printed composite Specimen T230 containing different types of voids.

Due to the improper impregnation between resin and fiber and the lack of consolidation pressure, porosity or void is a typical manufacturing defect of printed composite structures. As illustrated in Figure 13, voids can basically be divided into two categories based on their spatial distribution: (i) intra-bead voids: the voids mainly distributed inside the fiber bundles, indicated by the red lines, which is caused by the incomplete impregnation between the thermoplastic resin and the fibers; and (ii) inner-bead voids: the voids mainly distributed in between the printed filaments, indicated by the blue lines, which is caused by the insufficient flow of the pre-deposited filament due to the cold filament interface combined with low consolidation pressure [20].

**Figure 14:**
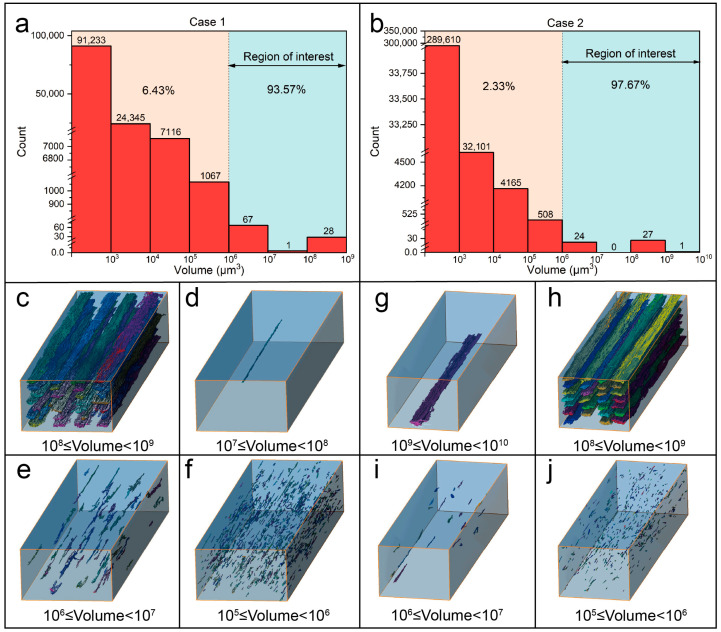
Statistical diagram of the number of voids at different volume levels within two representative printed specimens: (**a**) T230 and (**b**) H0.4. Three-dimensional reconstructions and visualization of voids for the Specimen T230 with different volume ranges: (**c**) 1 × 10^8^~1 × 10^9^ μm^3^, (**d**) 1 × 10^7^~1 × 10^8^ μm^3^, (**e**) 1 × 10^6^~1 × 10^7^ μm^3^, and (**f**) 1 × 10^5^~1 × 10^6^ μm^3^. Three-dimensional reconstructions and visualization of voids for the Specimen H0.4 with different volume ranges: (**g**) 1 × 10^9^~1 × 10^10^ μm^3^, (**h**) 1 × 10^8^~1 × 10^9^ μm^3^, (**i**) 1 × 10^6^~1 × 10^7^ μm^3^, and (**j**) 1 × 10^5^~1 × 10^6^ μm^3^.

In this research, the printed samples were mainly divided into two categories according to the size of the maximum void volume: Case 1 indicates the samples with the maximum void volume in the range of 1 × 10^8^~1 × 10^9^ μm^3^, represented by the Specimen T230 (see Figure 14a); and Case 2 indicates the samples with the maximum void volume greater than or equal to 1 × 10^9^ μm^3^, represented by the Specimen H0.4 (see Figure 14b). Figure 14c–j illustrate the distribution of the voids with different volume magnitudes. Obviously, the voids with a volume range of 1 × 10^8^~1 × 10^9^ μm^3^ are all intra-bead voids, which are distributed alongside the printing direction. The voids with a volume range of 1 × 10^9^~1 × 10^10^ µm^3^ are a result of the connection of intra-bead voids and inter-bead voids. Other smaller voids (≤1 × 10^8^ μm^3^) are randomly distributed between the filaments, which therefore belong to inter-bead voids. As observed from Figure 14a,b, for both cases, the voids with volume size higher than 1 × 10^6^ μm^3^ account for the major proportion, higher than 93% of the entire void content inside the specimens. Therefore, the subsequent discussion will only focus on voids with a volume greater than or equal to 1 × 10^6^ μm^3^ since the rest part has little effect on the mechanical performance of the samples.

#### 3.2.1. Effect of Printing Parameters on Void Dimension

**Figure 15:**
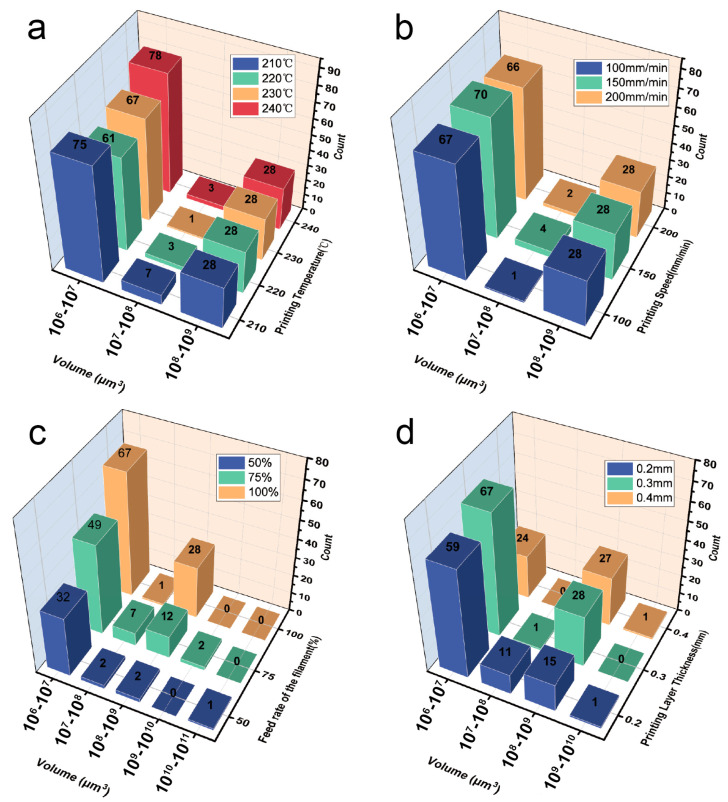
The bar chart statistics of the number of voids at different volume magnitudes based on the effects of different printing parameters: (**a**) printing temperature, (**b**) printing speed, (**c**) feed rate of the filament, and (**d**) layer thickness.

A quantitative statistic on the distribution of voids at different volume magnitudes as a function of different printing parameters is illustrated in Figure 15. It can be observed that the count of voids in the volume size of 1 × 10^8^~1 × 10^9^ μm^3^ is approximately 28, corresponding to the number of printed filaments. The printing temperature and printing speed barely have any effect on the distribution of void numbers at different volume levels (see Figure 15a,b). On the contrary, when the feed rate of the filament decreases, voids with larger volume segments appear, and the number of voids within the range of 1 × 10^8^~1 × 10^9^ μm^3^ gradually decreases, which implies that the connection between the intra-bead voids and the inter-bead voids. A large void with a volume range of 1 × 10^10^~1 × 10^11^ μm^3^ was found once given a feed rate of the filament of 50%, which connected most filaments inside the specimen. Regarding Figure 15d, it is found that an obvious decrease in the voids within the range of 1 × 10^8^~1 × 10^9^ μm^3^ while an increase in the ones within 1 × 10^7^~1 × 10^8^ μm^3^ when given a decreased layer thickness (i.e., 0.2 mm), which is owing to the increased ironing pressure between the nozzle and the deposited filament [17].

#### 3.2.2. Porosity Distribution in X Direction

**Figure 16:**
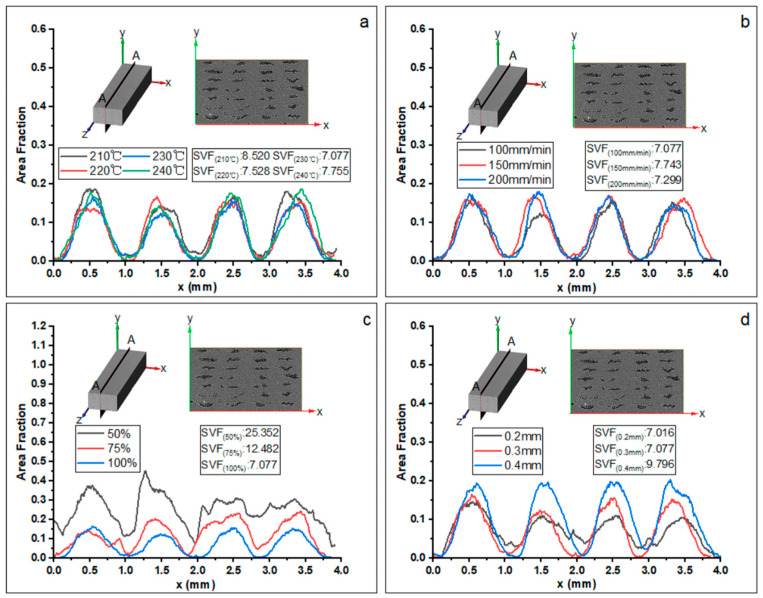
Evolution of the porosity content of the A-A plane slice along the *X*-axis with different printing parameters: (**a**) printing temperature, (**b**) printing speed, (**c**) feed rate of the filament, and (**d**) layer thickness. SVF represents the total porosity content inside the specimen within three-dimensional space. The lower corner mark represents the corresponding printing parameters.

In addition to analyzing the void distribution at different volume magnitudes, an investigation of the spatial distribution of the voids within the specimen was conducted. Figure 16 shows the void fraction of the printed specimen along the transverse direction. For simplification, the void contents at different directions are labeled with three different axes, i.e., X, Y, and Z axis (direction). One should note that the statistics index is the sum of the porosity of the A-A slice at the Y-Z plane, as indicated by the schematic in Figure 16a. Four peaks generally correspond to the central line of the four printed filament columns. The effects of printing temperature and printing speed on the porosity distribution are similar and insignificant; the maximum value is leveling at around 0.2, and the minimum value remains close to 0. A lower feed rate of the filament results in an apparent increase in both the maximum and minimum values of the void fraction, as illustrated in Figure 16c. The maximum values reach 0.3~0.4, and the minimum values are around 0.1~0.25 once given a feed rate of the filament of 50%. The increase in the maximum void fraction results from a higher dispersion of fibers, which is due to the reduction in internal pressure within the printing head and the less effective impregnation of the resin with the fiber [18], while the increase in the minimum values indicates the connection of the voids along the X direction, corresponding to the large void formation in the F50 Specimen (see Figure 15c), which can be explained by the lack of resin to fill in the gaps between different columns. Observing Figure 16d, the void content is increased when given a higher layer thickness, which is likely a consequence of the reduction in ironing force between the nozzle and the deposited filament [20].

#### 3.2.3. Porosity Distribution in Y Direction

**Figure 17:**
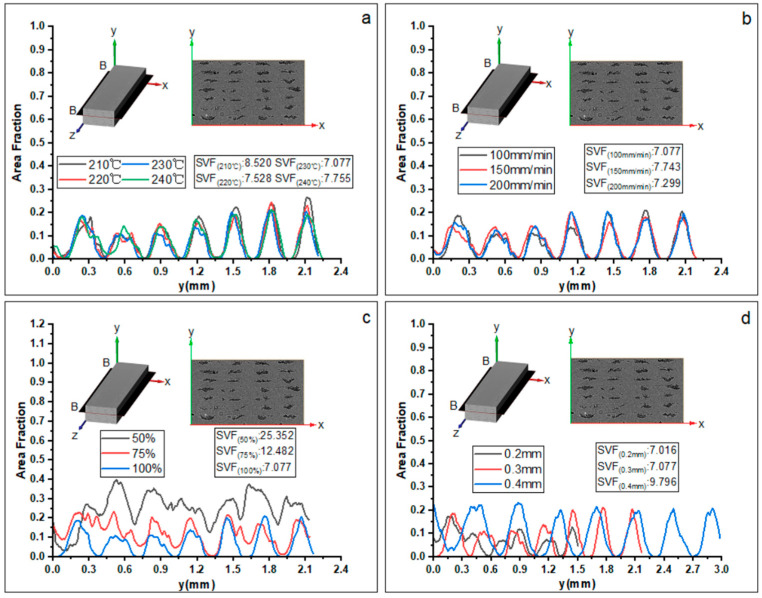
Evolution of the porosity content of the B-B plane slice along the *Y*-axis with different printing parameters: (**a**) printing temperature, (**b**) printing speed, (**c**) feed rate of the filament, and (**d**) layer thickness. SVF represents the total porosity content inside the specimen within three-dimensional space. The lower corner mark represents the corresponding printing parameters, and the statistical range is from the first layer to the seventh layer.

Figure 17 plots the evolution of the sum of porosity of the B-B slice at the X-Z plane with a varying thickness value in the Y direction. The curve peaks correspond to the number of layers. Differently, the peak values are located at 5/6-layer thickness from bottom to top, indicating the offset of the voids in the direction of each filament thickness. Figure 17a,b both show that the peak value decreases first and then increases gradually, and the positions of the second and third layers are the lowest, causing the initial print layers to be under continuous pressure until the end of printing. Similar to Figure 16, changing the printing temperature or printing speed has little effect on the porosity values in the Y direction. However, the changes in the feed rate of the filament and layer thickness have more significant impacts on the porosity distribution. When the feed rate of the filament decreases, both the maximum and minimum values of the curves increase, as shown in Figure 17c. Similar to the observations in Figure 16c, the maximum void fraction reaches 0.4 and the minimum void fraction reaches 0.2 when given a feed rate of the filament of 50%, indicating that the voids along the Y direction are no longer independent of each other. Figure 17d shows that when the layer thickness is 0.4 mm, the peak porosity values of different layers are basically the same. When the layer thickness is 0.2 mm, the maximum value decreases while the minimum one increases, which is similar to the observation in Figure 16d.

#### 3.2.4. Porosity Distribution in Z Direction

**Figure 18:**
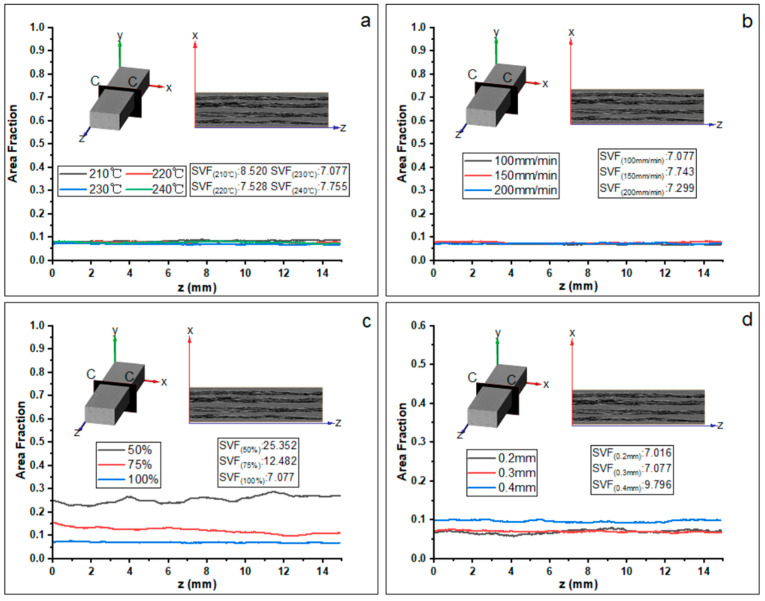
Evolution of the porosity content of the C-C plane slice along the *Z*-axis with different printing parameters: (**a**) printing temperature, (**b**) printing speed, (**c**) feed rate of the filament, and (**d**) layer thickness. SVF represents the total porosity content inside the specimen within three-dimensional space. The lower corner mark represents the corresponding printing parameters.

As shown in Figure 18, the curve describes the evolution of the porosity of the C-C slice at the X-Y plane with a varying value at the Z axis. Basically, the porosity is constantly leveling around 0.07 when given different printing temperatures (Figure 18a) and printing speeds (Figure 18b). Decreasing the feed rate of the filament significantly increases the porosity from 0.07 to 0.25, as illustrated in Figure 18c. Figure 18d shows that the porosity is maximum when the layer thickness is 0.4 mm.

## 4. Conclusions

In this paper, Micro-CT technology was used to quantitatively analyze the influence of several typical printing parameters on the filament morphologies and printing voids of 3D-printed carbon fiber-reinforced PLA composite specimens. The effects of different processing parameters, i.e., printing temperature, printing speed, feed rate of the filament, and layer thickness on the micro-structures of the printed specimens were systematically investigated. In comparison, the effects of printing temperature and printing speed were not significant. Some conclusion points are drawn:

1. Due to the geometric position of the fiber bundle and resin filament in the heating block and the inertial action during the printing processes, the fiber bundle was mainly distributed in the upper part of the deposited filament, which led to the offset distribution of the voids within the filament. The printing voids within the 3D-printed composite specimens can basically be divided into two types: intra-bead voids with a general volume of 1 × 10^8^~1 × 10^9^ μm^3^ and inter-bead voids with a volume normally less than 1 × 10^8^ μm^3^.
2. When the feed rate of the filament decreased, the filament shape was more likely to be the ideal shape. However, due to the weak constraints of resin, fibers could be distributed throughout the filament, which resulted in a high dispersion of the filament shape. Given a low layer thickness, the size of the filament was smaller than the theoretical shape, and the deposited filament was likely filled with pure resin due to the fracture of the fiber bundle.
3. The reduction in the feed rate of the filament led to an increase in both the maximum and minimum void values, while the decrease in the layer thickness caused an increase in the minimum value but a decrease in the maximum ones.
4. This study primarily focuses on the impact of the printing process on filament size and microscopic defects. However, the evaluation of mechanical properties under different printing parameters is lacking, which could be considered for further investigation in subsequent research.

## Figures and Tables

**Table 1:** Experimental design of the parametric analysis of the 3D printing process.

Specimen	T (°C)	S (mm/min)	F (%)	H (mm)
T210	**210**	100	100	0.3
T220	**220**	100	100	0.3
T230	**230**	100	100	0.3
T240	**240**	100	100	0.3
S150	230	**150**	100	0.3
S200	230	**200**	100	0.3
F75	230	100	**75**	0.3
F50	230	100	**50**	0.3
H0.2	230	100	100	**0.2**
H0.4	230	100	100	**0.4**

The bold part represents the printing parameters of interest, e.g., T210 represents printing temperature as the printing parameter of concern; 210 is the specific value.

## Data Availability

Not applicable.
